# Proteomic and epigenomic markers of sepsis-induced delirium (SID)

**DOI:** 10.3389/fmolb.2015.00059

**Published:** 2015-10-26

**Authors:** Adonis Sfera, Amy I. Price, Roberto Gradini, Michael Cummings, Carolina Osorio

**Affiliations:** ^1^Department of Psychiatry, Loma Linda UniversityLoma Linda, CA, USA; ^2^Psychiatry, Patton State HospitalPatton, CA, USA; ^3^Evidence Based Health Care, University of OxfordOxford, UK; ^4^Department of Pathology, Sapienza UniversityRome, Italy; ^5^IRCCS NeuromedPozzili, Italy

**Keywords:** Aquaporin-4 (AQP-4), astrocytes, cell cycle, lymphocyte proliferation test (LPT), T helper 17 cells, exosomes, microRNA

## Abstract

In elderly population sepsis is one of the leading causes of intensive care unit (ICU) admissions in the United States. Sepsis-induced delirium (SID) is the most frequent cause of delirium in ICU (Martin et al., 2010). Together delirium and SID represent under-recognized public health problems which place an increasing financial burden on the US health care system, currently estimated at 143–152 billion dollars per year (Leslie et al., 2008). The interest in SID was recently reignited as it was demonstrated that, contrary to prior beliefs, cognitive deficits induced by this condition may be irreversible and lead to dementia (Pandharipande et al., 2013; Brummel et al., 2014). Conversely, it is construed that diagnosing SID early or mitigating its full blown manifestations may preempt geriatric cognitive disorders. Biological markers specific for sepsis and SID would facilitate the development of potential therapies, monitor the disease process and at the same time enable elderly individuals to make better informed decisions regarding surgeries which may pose the risk of complications, including sepsis and delirium. This article proposes a battery of peripheral blood markers to be used for diagnostic and prognostic purposes in sepsis and SID. Though each individual marker may not be specific enough, we believe that together as a battery they may achieve the necessary accuracy to answer two important questions: who may be vulnerable to the development of sepsis, and who may develop SID and irreversible cognitive deficits following sepsis?

## Introduction

At present there are no biological markers to indicate vulnerability to sepsis or to the CNS dysfunction following it. Electroencephalography (EEG) and various instruments such as Confusion Assessment Method (CAM), Delirium Rating Scale (DRS) or Delirium Symptoms Interview (DSI) are currently used for diagnosing delirium, however they are not specific for SID and difficult to perform in sedated or intubated ICU patients (Zampieri et al., 2011). Recent studies have shown that delirium in general and SID in particular are unrecognized by clinicians in 32–66% of cases and because of their resemblance to psychiatric conditions, patients with delirium are frequently admitted to psychiatric wards, thus delaying much needed interventions (Leslie et al., 2008; Reeves et al., 2010). In order to avoid this problem the Society for Academic Emergency Medicine Geriatrics Task Force designated delirium screening a key quality indicator in elderly care (Terrell et al., 2009).

In our prior work in SID and delirium, we emphasized a potential epigenomic screening tool, the microRNA-6775 (miR-6775), which can be obtained from peripheral blood exosomes. This miR seems to block the transcription of CHRNA 7 gene which codes for alpha 7 nicotinic cholinergic receptors, while at the same time augments the expression of RNF-128, the Gene Related to Anergia in Lymphocytes (GRAIL), a gene involved in sepsis associated immune suppression (SAIS). Since SAIS represents a major cause of death in sepsis patients, miR-6775 may be a sepsis-stage marker in addition to a being a SID vulnerability screening tool (Sfera et al., 2014). We have hypothesized that delirium predisposing factors including low grade inflammation and the paucity of alpha 7 nicotinic acetylcholine receptors (nAChRs) may be the result of dysfunctional miR-6775 which silences the gene coding for these receptors. If this hypothesis is correct, miR-6775 may emerge as a marker of vulnerability to both sepsis and SID. In addition to epigenomic markers we drew attention to an astrocytic proteomic marker, the aquaporin-4 (AQP-4) protein which was found to be up-regulated in delirium (Sfera and Osorio, 2015).

In the present article, instead of focusing on individual biomarkers, we present a proteomic battery consisting of molecules released by the innate immune system, the initiator of septic responses. These markers can be obtained from the peripheral blood exosomes, a platform which carries to the periphery the molecular fingerprints of cells located in various tissues including the brain.

## From the immunological to the neurological synapse: Pathophysiology of sepsis and SID

Critical care physicians have known for a long time that 70–80% of patients diagnosed with severe sepsis had been admitted to the hospital for reasons other than infections (Munford and Suffredini, 2010). Indeed, infection is necessary for a septic response, but not sufficient, as most infections remain localized and the inflammation does not spread throughout the body tissues. Moreover, sepsis-like global inflammatory responses are known to occur in burns, trauma or pancreatitis in the absence of infection (Balk, 2014).

Sepsis is thought to be ignited by a failure of the innate immune system to turn “off” after initiating an initial inflammatory response to intruding pathogens. The innate immunity “on” and “off” switch is located at the immunological synapse, the nanoscale junction between the T lymphocytes and the antigen presenting cells (APCs). This junction is populated by pattern recognition receptors (PRR) out of which the toll-like receptors (TLRs) are the best known. Activation of TLRs by pathogen toxins triggers “cytokine storms,” release of non-specific pro-inflammatory molecules. Switching “off” the innate immune system is epigenomically controlled by microRNAs action on TLRs (Bayarsaihan, 2011; Stearns-Kurosawa et al., 2011). Turning the innate immunity “off” must occur when the more specific adaptive immune system comes “on line.” Sepsis may be triggered by defective microRNAs which may be incapable of deactivating the TLRs and turn the innate immunity “off.” In this scenario, both the adaptive and the innate immunity continue to work in parallel, eventually leading to immune exhaustion or SAIS.

MicroRNAs or miRs are short, 18–22 nucleotides in length, non-coding RNAs which have the ability to silence gene expression by inhibiting or degrading their messenger RNAs (mRNA). MicroRNAs were shown to modulate multiple biological processes, but those of interest for sepsis and SID include activation/deactivation of the innate immune system, cytoskeletal rearrangements in the innate immune cells (which enable cellular proliferation), and modulation of astrocytic volume (by changes in membrane permeability).

The CNS-immune interaction was demonstrated to be mediated by transmigration of peripheral lymphocytes and microphages through the blood-brain-barrier (BBB) and via the recently described lymphatic vessels in the dura mater (Louveau et al., 2015). The discovery of CNS lymphatics suddenly changed the perspective on the peripheral-central immune cooperation into a holistic one in which astrocytes, microglia and peripheral macrophages work in an innate immune continuum (Sternberg, 2006; Louveau et al., 2015). For example, the peripheral dendritic cells (DC) immigrate into the CNS and function as antigen presenting cells (APCs), while the activated microglia emigrate from the brain to the periphery, transporting inflammation (Sfera et al., 2014; Sfera and Osorio, 2015). These findings provide an explanation for the previously observed impairment in peripheral immunity following CNS pathology such as strokes, neurosurgical interventions, Alzheimer's disease (AD) and schizophrenia (Hochmeister et al., 2008; D'Agostino et al., 2012).

In the CNS, astrocytes and microglia are full members of the innate immune system which respond to peripheral infections by adopting a reactive stance (reactive gliosis). For example, reactive astrocytes undergo hypertrophy (edema) probably in an attempt to erect an additional physical barrier and restrict the spread of infection (Majewska and Szczepanik, 2006). Astrocytic swelling due to aquaporin-4 (AQP-4) up-regulation was documented in SID and may explain why most known delirium biomarkers are released by astrocytes (Papadopoulos and Verkman, 2013; Sfera et al., 2014; Thrane et al., 2014).

It is reasonable to assume that astrocytic swelling (astroedema) along with hypocholinergia (due to silenced CHRNA 7 gene) activate the TLRs, engendering low grade inflammation which is considered a delirium vulnerability marker in elderly (Court et al., 2001; Mitsis et al., 2009). For example, in post-operative delirium activation of TLR-4 (expressed by microglia and astrocytes) was demonstrated (Jalleh et al., 2012; Sofroniew, 2015). MicroRNA-130a is known to reduce astroedema by silencing the transcription of AQP-4 proteins (Sepramaniam et al., 2012). Moreover, miR-132 was demonstrated to restore CNS cholinergic signaling (Shaked et al., 2009). These epigenomic markers may represent a CNS compensatory response to astroedema and damaged cholinergic signaling.

## Proteomic biomarkers of SID

*Aquaporin-4 (AQP-4) proteins* in astrocytic end-feet constitute specific markers for astroedema described in SID. Exosomes carrying AQP-4 proteins from astrocytes to the peripheral blood may be utilized as biomarkers and therapeutic targets especially in SAIS (Papadopoulos and Verkman, 2013; Thrane et al., 2014; Sfera and Osorio, 2015).

*Galectin-9 (Gal-9)* and its receptor, the T cell immunoglobulin mucin-3 (Tim-3) expressed on T lymphocytes was shown to inhibit Th-17 proliferation both *in vitro* and *in vivo* (Oomizu et al., 2012). Gal-9 was demonstrated to play a major role in cell cycle regulation as it was shown to inhibit the cell cycle arrest in the G1 phase, promoting cellular proliferation. A dysfunctional Gal-9 may explain the aberrant neuronal cell cycle re-entry documented in AD as well as the exaggerated proliferation of macrophages and T-cells in the early phase of sepsis (Currais et al., 2009). In the CNS, Gal-9 is secreted by astrocytes and it was demonstrated to affect the astrocyte-microglia dialog (Steelman and Li, 2014). Interestingly, microRNA-155 (which modulates TLRs at the immunologic synapse) acts on the Tim-3 receptors, competing with Gal-9 (Cheng et al., 2015). Therefore, a significant drop in miR-155 level and/or an increase in Gal-9 level may suggest immune restoration in sepsis.

*CRYAB (alphaB-crystallin)* is a small heat-shock protein which was demonstrated to reduce T cell proliferation (including the CD-69 response). Animal studies show that Cryab-/- mice present with higher Th-17 cell count and more intense neuroinflammation, compared to wild-type counterparts (Arac et al., 2011). In the brain Cryab is secreted by astrocytes as an anti-inflammatory molecule; its presence in peripheral blood exosomes may indicate functional astrocytes, reflecting epigenetic integrity. CRYAB is involved in the process of microRNA maturation; its absence may result in dysfunctional microRNAs, including miR-6775, miR-155, miR-132, and miR-130 family. A drop in the level of this marker in peripheral blood exosomes may reflect the body-wide inflammation of sepsis with the imminent potential for SID onset (Ousman et al., 2007).

*Ubiquitin-modifying protein A 20* has potent anti-inflammatory and anti-proliferative properties. For example it can decrease T cell differentiation into the neurotoxic Th-17 phenotype (Kool et al., 2011). Animal studies demonstrate that A 20 deficiency causes spontaneous neuroinflammation with reactive gliosis. In addition, it was demonstrated that that the A 20 protein deactivates the innate immunity at the level of the TLRs in the immunological synapse (Boone et al., 2004). Absence of TLR deactivation was demonstrated to result in exaggerated inflammatory responses, the “cytokine storms” characteristic of early sepsis. Micro RNA-155 epigenetically modulates the “on” and “off” activation of TLRs, suggesting involvement in sepsis and SID (O'Neill et al., 2011; Table 1). A drop in A 20 level along with an increase in miR-155 levels are markers of poor prognosis in sepsis and SID.

**Table 1:** **Potential proteomic markers for sepsis and SID**.

**Protein**	**Action**	**Effect**
AQP-4	-Augments astrocytic water permeability -Promotes astroedema	Detrimental for SID
Galectin-9	-Inhibits Th-17 proliferation	Beneficial for sepsis/SID
CRYAB	-Decreases CD-69 lymphocyte proliferation -Promotes microRNA maturation	Beneficial in sepsis/SID
A 20 protein	-Deactivates TLRs -Turns innate immunity “off ”	Beneficial in sepsis/SID
Vimentin	-Enables cytoskeleton for motility and proliferation	Detrimental in sepsis/SID

*Vimentin* is a major constituent of the cellular cytoskeleton which was found to orchestrate the Th-17 and possibly cancer cells migration into the CNS (Nieminen et al., 2006). Indeed, in sepsis an elevated Th-17 count was reported as vimentin equips T lymphocytes with both the motility and the amoeboid shape necessary for transmigration across the blood-brain-barrier (Nieminen et al., 2006). By rearranging the cytoskeleton vimentin facilitates proliferation of CD-69 lymphocyte in response to mitogens; this response can be measured by the lymphocyte proliferation test (LPT) (Nieminen et al., 2006; Ward et al., 2008). LPT is currently being used as a peripheral blood marker for AD and traumatic brain injury (TBI), based on the observation that lymphocytes derived from patients with AD present with a proliferation defect, a lower CD-69 count after mitogenic stimulation, compared to lymphocytes derived from normal individuals (Zhang et al., 2013). In fact the transition between elevated and depressed CD-69 response, may mark the precise point in time when a localized infection transitions to sepsis and SID. This transition may be captured by the stimulation index (SI) which represents the ratio of CD-69 expression level/endogenous CD-69 level. The SI may mirror the onset and progression of immune suppression and if obtained daily in ICU patients may detect sepsis onset. At the epigenetic level the CD-69 vimentin-induced response is modulated by miR-155 and miR-130 family, again rendering these miRs epigenetic markers for SID.

## Epigenomic biomarkers of SID

Approximately 60% of known miRs are expressed in the brain, rendering them almost ideal peripheral biomarkers for many CNS disorders (Song and Lee, 2015). Exosome-isolated miRs provide a higher diagnostic precision than miRs obtained from plasma (Stenvang et al., 2012).

*MicroRNA-6775:* silences the transcription of CHRNA-7 gene which codes for alpha 7 nicotinic acetylcholine receptors (nAChRs). When silenced CHRNA-7 gene augments the proliferation of Th-17 lymphocytes. Another action of microRNA-6775 involves activation of RNA-128, the gene related to anergia in lymphocytes (GRAIL), predisposing to SAIS (Sfera et al., 2014).

*MicroRNA-130 family*: nicknamed astromiRs, this miR family are modulators of cellular motility (Tu et al., 2014). Members of this family include:

*MicroRNA–130b*: promotes metastatic cancer aggressiveness by augmenting vimentin-induced cytoskeletal changes. MicroRNA-130b is believed to be an oncomiR as it augments malignant aggression by enhancing cellular ability to migrate. We however characterize miR-130b as a motilimiR since metastasizing is occurs secondary to increased cellular motility. By the same mechanism miR-130b may promote Th-17 transmigration into the CNS (Table 1). For this reason miR-130b can be considered both a therapeutic target and a biomarker of SID (Leavy, 2010).

*MicroRNA-130a* (miR-130a): may also be a therapeutic target in addition to being a SID biomarker This miR, silences the transcription of AQP-4 proteins in astrocytic end-feet, reducing astroedema and neuroinflammation (Sepramaniam et al., 2012). In addition, miR-130a inhibits cellular locomotion and proliferation and is being credited with inhibition of metastatic spread. Since its action opposes miR-130b, we consider it an antimiR to 130a (Murugaiyan et al., 2011).

*MicroRNA-155* (miR-155): In sepsis, a dysregulated miR-155 was shown to unleash inflammation via activation of TLRs preventing deactivation of the innate immune system upon activation of adaptive immunity. Others have shown that silencing miR-155 may be beneficial in experimental autoimmune encephalomyelitis (Murugaiyan et al., 2011), AD (Song and Lee, 2015), and other neuropsychiatric disorders (Hu et al., 2013). We believe that miR-155 is a plausible therapeutic target in SID in addition of being of diagnostic and prognostic benefit.

*MicroRNA-132*: acts as a natural cholinesterase inhibitor (also called a cholinomiR) as it enhances cholinergic signaling by inhibiting its degrading enzymes (Shaked et al., 2009; Nadorp and Soreq, 2014) (Table 2). Deficient acetylcholine is one of the best documented hypotheses of delirium. Daily levels of miR-132 obtained from peripheral blood exosomes in ICU patients with SID may reflect the prognostic trend.

**Table 2:** **Potential epigenomic markers of sepsis and SID**.

**MicroRNA**	**Action**	**Effect**
mir-6775	-Silences CHRNA-7 gene -Augments RNF-128	Detrimental for sepsis/SID Promotes low grade inflammation and SAIS
miR-130 a	-Silences transcription of AQP-4 gene -Decreases cell motility and proliferation (lowers CD-69 and Th-17 lymphocytes)	Beneficial sepsis/SID Lowers astroedema and Th-17 transmigration
miR-130b	-Increases cell motility and proliferation	
miR-155	-Increases CD-69 response -Activated TLRs by binding to galectin-9 receptor -Turns innate immunity “on”	Detrimental for sepsis/SID
miR-132	-Acetylcholinesterase inhibition -Restores cholinergic transmission	Beneficial for sepsis/SID

## Tiny bubbles: An exosomal transportation platform

Extracellular vesicles are tiny membrane structures which are divided by their size, into ectosomes, larger vesicles (over 100 nm), and exosomes, smaller vesicles (50–100 nm). They are released by a wide variety of cells including immune, CNS and cancer cells. Their cargo consists of proteins, DNA, microRNAs, and mRNAs which carry the signatures of their cells of origin throughout the body fluids. After release from their source, miRs circulate with the peripheral blood either bound to lipoproteins or incorporated in exosomes which offer protection from dilution and the ambient ribonuclease (Wagner et al., 2013). Therefore, assays of exosome-packaged miRs derived from the peripheral blood are more reliable compared to assays of non-exosomal miRs (Cheng et al., 2014). Exosome content can be analyzed with the help of simple commercial exosome isolation kits or by more complex ultracentrifugation (Corrado et al., 2013).

At the immunological synapse, exosomes carry pathogen-derived molecules between the T cell and the APC, committing T cells to particular phenotypes (Schorey et al., 2015). In sepsis it was demonstrated that naïve T cells differentiate into neurotoxic Th-17 lymphocytes (Martinez et al., 2008). We believe that this metamorphosis is enabled by the disinhibition of RNF-128 gene by dysfunctional miR-6775 which, probably acts on vimentin to enable transformation of naïve T cells into the highly mobile and aggressive Th-17 phenotype.

In the CNS, exosomes were demonstrated to be involved in neurotransmitter signaling at neurological synapses (Chivet et al., 2014; Glebov et al., 2015). For example, exosomal miRs in the prefrontal cortex synapses were recently demonstrated in schizophrenia and bipolar disorder (Banigan et al., 2013). In SID exosome-analysis may identify stage-of-disease markers and treatment targets. For example, astrocytes were shown to release exosomes containing miR-155 and miR-130 family which along with AQP-4 proteins which constitute SID biomarkers. Indeed the National Institute of Health recently awarded a grant for studying blood exosomes containing astrocytic proteins as diagnostic markers for CNS disorders (Crocker and Vella, 2015). In AD neural proteins such as amyloid beta, total tau and P-tau were recently isolated from peripheral blood exosomes (Fiandaca et al., 2015).

Exosomes were demonstrated capable of carry aquaporin proteins to the body periphery. For example, urinary exosomes containing aquaporin-1 (AQP-1) proteins are currently being utilized in nephrology as novel biomarkers of renal ischemia and reperfusion (Sonoda et al., 2009). In SID, the up-regulated AQP-4 proteins originating in astrocytic end-feet may be detectable in peripheral blood exosomes.

## Conclusions

Sepsis and SID represent health care problems which translate into a major economic burden likely to increase along with the demographics of aging population. Biological markers with adequate sensitivity and specificity for SID could decrease not only the length of hospital stay, lead to new treatments, but also preempt the epidemic of irreversible cognitive deficit and dementia in elderly. With the same token the ability to predict vulnerability to delirium and subsequent dementia would facilitate the informed consent process of elderly individuals deciding for elective surgeries.

### Conflict of interest statement

The authors declare that the research was conducted in the absence of any commercial or financial relationships that could be construed as a potential conflict of interest.
